# Capacity Assessment Training and Competency Evaluation Tool

**DOI:** 10.1093/geroni/igaa057.1998

**Published:** 2020-12-16

**Authors:** Lindsey Jacobs, Patricia Bamonti, Jessica Strong, Kyle Page, Barry Edelstein, Rebecca Allen, Shane Bush

**Affiliations:** 1 VA Boston Healthcare System, Brockton, Massachusetts, United States; 2 University of Prince Edward Island, Charlottetown, Prince Edward Island, Canada; 3 Edward Hines, Jr. VA Hospital, Hines, Illinois, United States; 4 WVU, Morgantown, West Virginia, United States; 5 Alabama Research Institute on Aging, Tuscaloosa, Alabama, United States; 6 University of Alabama, Tuscaloosa, Alabama, United States

## Abstract

Given the complex interplay of ethical, clinical, and legal factors, evaluating capacities in older adults is an important competency for geropsychologists. However, the amount of quality of training in this area varies, and geropsychology trainees report less confidence in their capacity evaluation skills. To date, only the Pikes Peak Self-Assessment Tool includes items measuring competency and growth in decisional capacity evaluations. However, it is a broad self-report measure assessing general geropsychology competencies. We developed a performance-based measure of decision-making capacity evaluations, the “Capacity Assessment Training and Competency Evaluation Tool (CATCET).” Using the ABA/APA Assessment of Older Adults with Diminished Capacity as a guide, expert panels created two clinical cases across 5 capacity domains. This presentation will discuss the creation of the CATCET, its application as a training and evaluation tool, and initial performance data among psychology graduate students, intern, and fellows across settings.

